# Advantages of phylogenetic distance based constrained ordination analyses for the examination of microbial communities

**DOI:** 10.1038/s41598-017-06693-z

**Published:** 2017-07-25

**Authors:** V. Shankar, R. Agans, O. Paliy

**Affiliations:** Department of Biochemistry and Molecular Biology, Boonshoft School of Medicine, Wright State University, Dayton, Ohio USA

## Abstract

Recently developed high throughput molecular techniques such as massively parallel sequencing and phylogenetic microarrays generate vast datasets providing insights into microbial community structure and function. Because of the high dimensionality of these datasets, multivariate ordination analyses are often employed to examine such data. Here, we show how the use of phylogenetic distance based redundancy analysis provides ecological interpretation of microbial community differences. We also extend the previously developed method of principal response curves to incorporate phylogenetic distance measure, and we demonstrate the improved ability of this approach to provide ecologically relevant insights into temporal alterations of microbial communities.

## Introduction

Recent advances in high-throughput massively parallel sequencing and phylogenetic microarrays have led to a bloom of studies in the field of microbial ecology^1, 2^. Because these experimental platforms generate large datasets of measured values such as sequence read counts or microarray probe hybridization signals, multivariate statistical analyses are usually employed to interpret the acquired data^3, 4^. Unconstrained ordination analyses such as principal coordinate and component analyses are frequently used to assess the variability in the datasets and distribute samples in lower dimensional space according to the matrix of measured values. While useful, these exploratory techniques do not provide a direct assessment of how different explanatory variables such as environmental gradients, sample groups, or patient metadata (age, weight, gender, etc) contribute to the observed patterns in microbial community variability. To provide such assessment, the use of constrained methods is advocated^3^. The two widely used approaches, redundancy analysis (RDA) and canonical correspondence analysis (CCA), utilize Euclidean and χ^2^distances, respectively, to calculate the relationships among samples^5, 6^. Neither distance measure takes into consideration the phylogenetic makeup of microbial communities and thus is not able to take advantage of this ecologically important information. Phylogenetic trees closely resemble clusters obtained on the basis of shared gene content^7^, and the microbial phylogeny and function were shown to be linked for many microbial traits^8, 9^ (note however that significant variability in gene content and functional capacity can often exist even among different strains and closely related species^10^). Thus, the microbial community composition and function are dependent to at least some degree on the phylogeny of its members^11, 12^. In this report we show that phylogenetic distance based constrained analyses provide ecological interpretation of microbial community datasets.

## Results and Discussion

**Figure 1:**
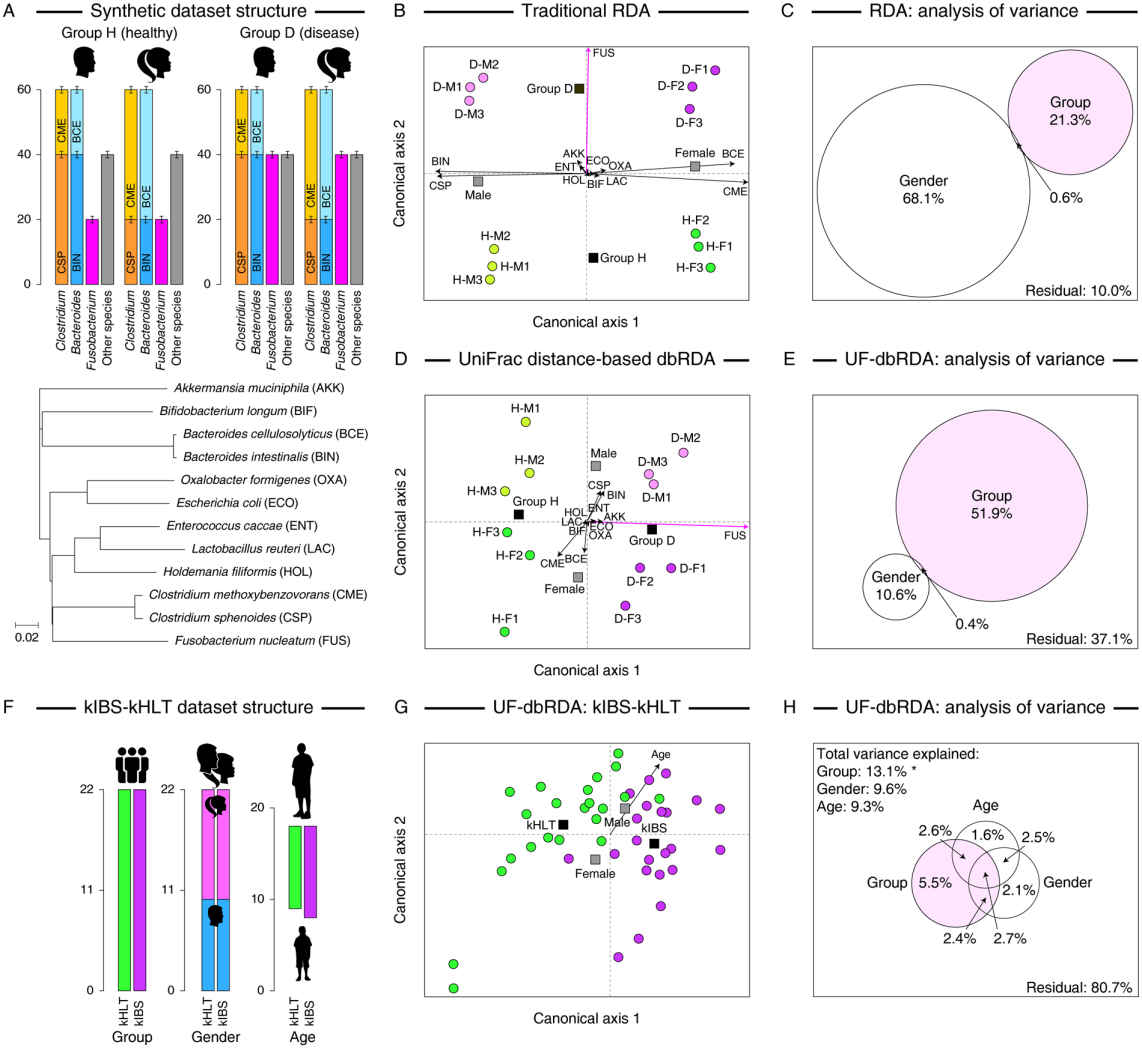
Comparison of the outputs between RDA and weighted UniFrac distance based dbRDA. (**A**) Structure of synthetic community dataset used as input for RDA ordination analyses. Top panel shows the differences between groups in the abundances of community members; bottom panel depicts the phylogenetic relationship among species. (**B**) and (**D**) Triplots of the Euclidean distance-based RDA output (panel **B**) and the weighted UniFrac distance-based RDA output (panel **D**). First two canonical axes are visualized. Species scores are shown as arrows; species names are shown in three-letter code (please refer to phylogenetic tree in panel **A** for definitions). Explanatory variables are shown as squares; samples are shown as colored circles. Sample names designate group (“D” or “H”) and “gender” (M” or “F”). (**C**) and (**E**) Venn diagrams present the analysis of variance of RDA (panel **C**) and weighted UniFrac distance-based RDA (panel **E**) models. Structure (panel **F**), UF-dbRDA ordination output (panel **G**), and analysis of variance of UF-dbRDA model (panel **H**) of the kIBS-kHLT dataset originally published by Rigsbee *et al*.^22^. In panel **H**, (*)indicates a statistically significant relationship between an explanatory variable and the response variable dataset at α = 0.01 level.

To illustrate the advantage of phylogenetic distance based constrained ordination in microbial ecology, we extended the use of a distance-based variant of redundancy analysis (dbRDA)^13, 14^ to utilize the phylogenetic UniFrac (UF) distance-based matrix of (dis)similarities among samples in a dataset as has been done in several previous studies^15–18^. UniFrac distance is computed by calculating the fraction of branch lengths of a combined phylogenetic tree that are not shared between two communities^19^. Thus, two communities that mostly have members of phylogenetically distinct clades would have large a UF distance, whereas communities that consist of different members of the same phylogenetic clade (e.g., same genus but different species) would have a small UF distance. By incorporating the abundance estimates of each taxon in different samples, a weighted UniFrac measure (wUF) can also be calculated. To compare the performance of wUF-dbRDA with several other commonly used constrained ordination analysis including Euclidean and Bray-Curtis distance-based RDAs as well as CCA, we designed a small synthetic microbial community consisting of a dozen bacterial members known to reside in the human gastrointestinal tract. We chose to use a synthetic dataset at this stage over an actual example of microbial community in order to reduce overall dataset variance and limit noise arising due to many low-abundance members typically present in most microbial communities^20^. Response variables were counts of each species’ abundance simulated manually with random noise (±10% and ±20% of each species target level for more and less abundant species, respectively) as shown in Fig. 1A. Two explanatory variables were defined, “group” and “gender”. Group variable contained two choices, either samples were drawn from (i) “healthy” patients (group H), or (ii) patients with a “disease” (group D). The groups differed by two-fold in the abundance of *Fusobacterium nucleatum*, a gut bacterial species that has been associated with human colorectal cancer^21^. Within each group, we introduced further dichotomy between “genders” by varying which species of *Clostridium* and *Bacteroides* they harbored (Fig. 1A). The overall abundance of each of these two genera did not differ between genders, and thus phylogenetically there is little overall distinction between “male” and “female” samples. The full numerical dataset is provided in Supplementary File 1. The outputs (first two canonical axes) of traditional (Euclidean) RDA and wUF-dbRDA ordination analyses of this synthetic dataset are visualized in Fig. 1B,D, respectively, whereas the analysis of dataset variance is shown in Fig. 1C,E. Because RDA weighs the importance of all variables equally and only takes into consideration their numerical values, it separates equally well both H/D groups and genders in the constrained ordination space, and reveals significant contribution of variation in *Clostridium* and *Bacteroides* members towards overall data variability. This is evident by the canonical axis 1 separating genders rather than H/D groups (Fig. 1B), and because two-thirds of the overall variance in the synthetic microbial dataset were attributed to the “gender” explanatory variable (Fig. 1C). Both canonical correspondence analysis as well as Bray-Curtis distance based dbRDA analysis applied to the same dataset produced the outputs similar to that of traditional RDA (Supplementary Figure 1). In contrast, phylogenetic distance based wUF-dbRDA attributes much less variance to gender (11% vs 68%, see Fig. 1E) and instead separates samples first according to health/disease status (canonical axis 1 in Fig. 1D). Thus, while wUF-dbRDA reveals *Fusobacterium* as the driver of microbial community differences between H and D cohorts as was designed in the structure of our synthetic dataset, that finding is less prominent in traditional RDA and CCA analyses which focus more on the phylogenetically minor variation within *Bacteroides* and *Clostridium* genera.

We subsequently applied UF-dbRDA analysis to the microbiota abundance dataset available from the Rigsbee *et al*. study^22^. The dataset comprised phylogenetic microarray based abundance values for 775 phylotypes of human gut microbiota profiled in two cohorts of teenagers: healthy group (designated kHLT) and those diagnosed with diarrhea-predominant irritable bowel syndrome (designated kIBS). Group (healthy vs IBS), gender, and age served as explanatory variables in our UF-dbRDA analysis (see Fig. 1F). The UF-dbRDA ordination using the first two canonical axes is shown in Fig. 1G, and the analysis of variance is presented in Fig. 1H. While there were many unknown gradients of variance influencing the microbiota composition (expected for the complex human gut microbiota dataset and indicated by a large fraction of residual unexplained variance), the group assignment was the most dominant predictor among the explanatory variables tested. It accounted for the largest explained variance (Fig. 1H), the samples were separated according to the group assignment along the first canonical axis (Fig. 1G), and it was the only statistically significant relationship between explanatory and response variables. Unconstrained principal coordinates analysis of the same dataset similarly indicated a visual separation of kIBS and kHLT samples in the ordination space, though it could not provide statistical evaluation of the relationship (see ref. 23).

Phylogenetic distance-based redundancy analysis has also been successfully employed in other recent studies, where dbRDA was used to reveal the extent to which age, body mass index, and country of residence influenced gut microbiota composition in US and Egyptian teenagers^18^, to uncover major genera driving skin microbiome differentiation among individuals^17^, to test the separation of gut microbiota of IBS patients from that of healthy controls^15^, to show the effects of plant host, amount of available nitrogen, and competitor removal on the root-associated bacterial community assembly^16^, to test if cloacal microbiome of barn swallows differed between males and females and between breeding colonies^24^, and to identify the major environmental factors controlling bacterial and fungal community composition in soils^25–27^.

**Figure 2:**
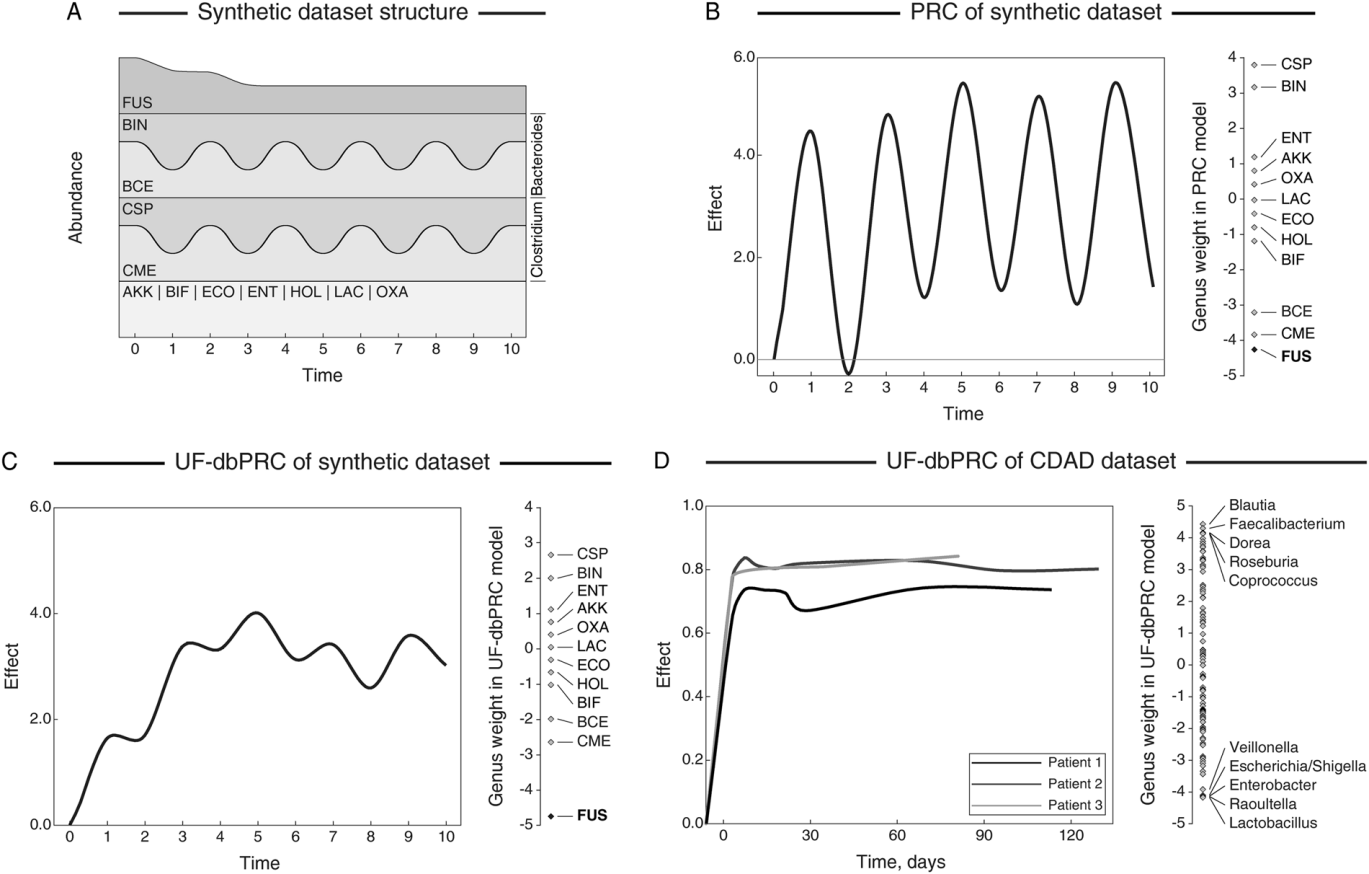
Comparison of the outputs between PRC and weighted UniFrac distance based dbPRC. (**A**) Structure of synthetic community dataset used as input for PRC ordination analyses. Please refer to phylogenetic tree in Fig. 1A for definitions of species codes. (**B**) and (**C**) Principal response curves plots for PRC (panel **B**) and weighted UniFrac distance-based dbPRC (panel **C**) analyses. Genus weights contributing to each statistical model are shown on the right side of each panel. (**D**) wUF-dbPRC analysis of genus level community structure in three patients with *Clostridium difficile* associated disease (CDAD) undergoing fecal microbiota transplantation (original dataset was published by Shankar *et al*.^29^). Each curve corresponds to a different individual as shown; genus weights are provided in the right side panel. Initial time point corresponds to community structure prior to FMT, all other time points represent days after FMT procedure, which was carried out at time 0.

To further demonstrate the utility of phylogenetic distance based constrained ordination analyses, we also extended the method of principal response curves (PRC)^28^ to use a phylogenetic distance measure in its calculations. PRC was originally developed to analyze time-series data and carries out partial RDA ordination to obtain estimates of community changes using time as a predictor variable. Here, we developed an extension of PRC by incorporating phylogenetic weighted UniFrac distance into its distance matrix calculations. We compared the performance of wUF-dbPRC and Euclidean distance-based PRC on a synthetic community dataset visualized in Fig. 2A. The dataset contained abundance values for the same set of 12 bacterial species shown in Fig. 1A, and incorporated a gradual reduction of *Fusobacterium* abundance over the observation period. Abundances of individual species of *Bacteroides* and *Clostridium* oscillated from one time point to another; however, the overall abundance of each of these genera remained the same (see Fig. 2A). The full numerical dataset is provided in Supplementary File 2. Standard PRC analysis showed a significant oscillating pattern in community structure, with no indication of consistent community alteration over time (Fig. 2B). Bray-Curtis distance based dbPRC analysis also showed an oscillating composition of the community (Supplementary Figure 2). In contrast, wUF-dbPRC output clearly revealed a community change starting from time point 1 and demonstrated that *Fusobacterium* is the main single driver of these changes (Fig. 2C).

We then applied the wUF-dbPRC analysis to the time-series measurements of microbiota composition taken from the study by Shankar and co-workers^29^. The study described fecal microbiota changes in three patients with *Clostridium difficile* associated disease following fecal microbiota transplantation (FMT) from a healthy donor. The results of the wUF-dbPRC analysis are presented in Fig. 2D. The fecal microbiota in all three patients changed drastically within few days following FMT procedure, and community remained stable over a three-month period. The analysis of variable weights from the wUF-dbRDA model identified the genera that contributed most to these changes (aerotolerant microbes decreased, many well-known fiber degraders increased in abundance following fecal microbiota transfer). These results match those originally reported in the Shankar *et al*. study based on the K-means cluster analysis^29^, and additionally provide the ability to quantitatively establish the main determinants of community alterations.

While our synthetic datasets were designed specifically to show the potential differences in outputs between traditional and weighted UniFrac distance-based RDA and PRC analyses, the above comparisons provide compelling evidence for the advantages of phylogenetic distance based constrained ordination analyses in the study of microbial communities.

## Methods

The constrained ordination techniques were performed in R using the *vegan* package^30^. Specifically, distance-based redundancy analysis was performed using the *vegan* function *capscale*. Principal response curves analysis was performed using the *prc* function. Analysis of variance was performed using the *anova.cca* command. The R code to run both dbRDA and dbPRC analyses is provided in Supplementary File 3; identical output for dbRDA can also be obtained with built-in Phyloseq R package functions.

## Electronic supplementary material

Supplementary  Information

## References

[1] Paliy O (2013). The golden age of molecular ecology. J Phylogen Evolution Biol.

[2] Xu JP (2006). Microbial ecology in the age of genomics and metagenomics: concepts, tools, and recent advances. Molecular ecology.

[3] Paliy O, Shankar V (2016). Application of multivariate statistical techniques in microbial ecology. Molecular ecology.

[4] Ramette A (2007). Multivariate analyses in microbial ecology. FEMS Microbiol Ecol.

[5] Amaral-Zettler LA (2011). Microbial community structure across the tree of life in the extreme Rio Tinto. Isme J.

[6] Jalanka-Tuovinen J (2013). Faecal microbiota composition and host-microbe cross-talk following gastroenteritis and in postinfectious irritable bowel syndrome. Gut.

[7] Zaneveld JR, Lozupone C, Gordon JI, Knight R (2010). Ribosomal RNA diversity predicts genome diversity in gut bacteria and their relatives. Nucleic Acids Research.

[8] Langille MG (2013). Predictive functional profiling of microbial communities using 16S rRNA marker gene sequences. Nat Biotechnol.

[9] Martiny, J. B. H., Jones, S. E., Lennon, J. T. & Martiny, A. C. Microbiomes in light of traits: A phylogenetic perspective. *Science* **350** (2015). 10.1126/science.aac9323 26542581

[10] Land M (2015). Insights from 20 years of bacterial genome sequencing. Funct Integr Genomic.

[11] Lozupone CA, Knight R (2008). Species divergence and the measurement of microbial diversity. Fems Microbiol Rev.

[12] Graham CH, Fine PV (2008). Phylogenetic beta diversity: linking ecological and evolutionary processes across space in time. Ecology letters.

[13] Legendre P, Anderson MJ (1999). Distance-based redundancy analysis: Testing multispecies responses in multifactorial ecological experiments. Ecol Monogr.

[14] Anderson MJ, Willis TJ (2003). Canonical analysis of principal coordinates: A useful method of constrained ordination for ecology. Ecology.

[15] Pozuelo, M. *et al*. Reduction of butyrate- and methane-producing microorganisms in patients with Irritable Bowel Syndrome. *Scientific Reports* **5** (2015). 10.1038/srep12693 PMC4523847 26239401

[16] Dean SL, Farrer EC, Porras-Alfaro A, Suding KN, Sinsabaugh RL (2015). Assembly of root-associated bacteria communities: interactions between abiotic and biotic factors. Env Microbiol Rep.

[17] Leung, M. H. Y., Wilkins, D. & Lee, P. K. H. Insights into the pan-microbiome: skin microbial communities of Chinese individuals differ from other racial groups. *Scientific Reports* **5** (2015). 10.1038/srep11845 PMC4503953 26177982

[18] Shankar V (2017). Differences in Gut Metabolites and Microbial Composition and Functions between Egyptian and U.S. Children Are Consistent with Their Diets. mSystems.

[19] Lozupone C, Knight R (2005). UniFrac: a new phylogenetic method for comparing microbial communities. Appl Environ Microbiol.

[20] Agans R (2011). Distal gut microbiota of adolescent children is different from that of adults. FEMS Microbiol Ecol.

[21] Castellarin M (2012). Fusobacterium nucleatum infection is prevalent in human colorectal carcinoma. Genome Res.

[22] Rigsbee L (2012). Quantitative profiling of gut microbiota of children with diarrhea-predominant Irritable Bowel Syndrome. Am J Gastroenterol.

[23] Shankar V, Agans R, Holmes B, Raymer M, Paliy O (2013). Do gut microbial communities differ in pediatric IBS and health?. Gut microbes.

[24] Kreisinger, J., Cizkova, D., Kropackova, L. & Albrecht, T. Cloacal Microbiome Structure in a Long-Distance Migratory Bird Assessed Using Deep 16sRNA Pyrosequencing. *PLoS ONE* **10** (2015). 10.1371/journal.pone.0137401 PMC4567286 26360776

[25] Goldmann, K. *et al*. Divergent habitat filtering of root and soil fungal communities in temperate beech forests. *Scientific Reports* **6** (2016). 10.1038/srep31439 PMC4980589 27511465

[26] Tian, J. *et al*. Patterns and drivers of fungal diversity along an altitudinal gradient on Mount Gongga, China. *Journal of Soils and Sediments*, 1–10, doi:10.1007/s11368-017-1701-9 (2017).

[27] Kim, M. *et al*. Highly Heterogeneous Soil Bacterial Communities around Terra Nova Bay of Northern Victoria Land, Antarctica. *PLoS ONE* **10** (2015). 10.1371/journal.pone.0119966 PMC4370865 25799273

[28] van den Brink PJ, ter Braak CJF (1999). Principal response curves: Analysis of time-dependent multivariate responses of biological community to stress. Environ Toxicol Chem.

[29] Shankar V (2014). Species and genus level resolution analysis of gut microbiota in Clostridium difficile patients following fecal microbiota transplantation. Microbiome.

[30] Oksanen, J. *et al*. Package ‘vegan’. *Community Ecology Package, version 2.9* (2013).

