# Supplementary material for: Advantages of phylogenetic distance based constrained ordination analyses for the examination of microbial communities
Source: Sci Rep. 2017 Jul 25;7:6481. doi: 10.1038/s41598-017-06693-z (PMC5526943; doi:10.1038/s41598-017-06693-z)
Supplement: Supplementary file 1 — Supplementary Information [file 41598_2017_6693_MOESM1_ESM.pdf]

Advantages of phylogenetic distance based constrained ordination analyses for the examination of microbial communities

Authors: V. Shankar, R. Agans, and O. Paliy*

SUPPLEMENTARY MATERIALS

Supplementary Figure 1: Canonical correspondence analysis and Bray-Curtis based Redundancy analysis performed on data from Supplementary File 1.

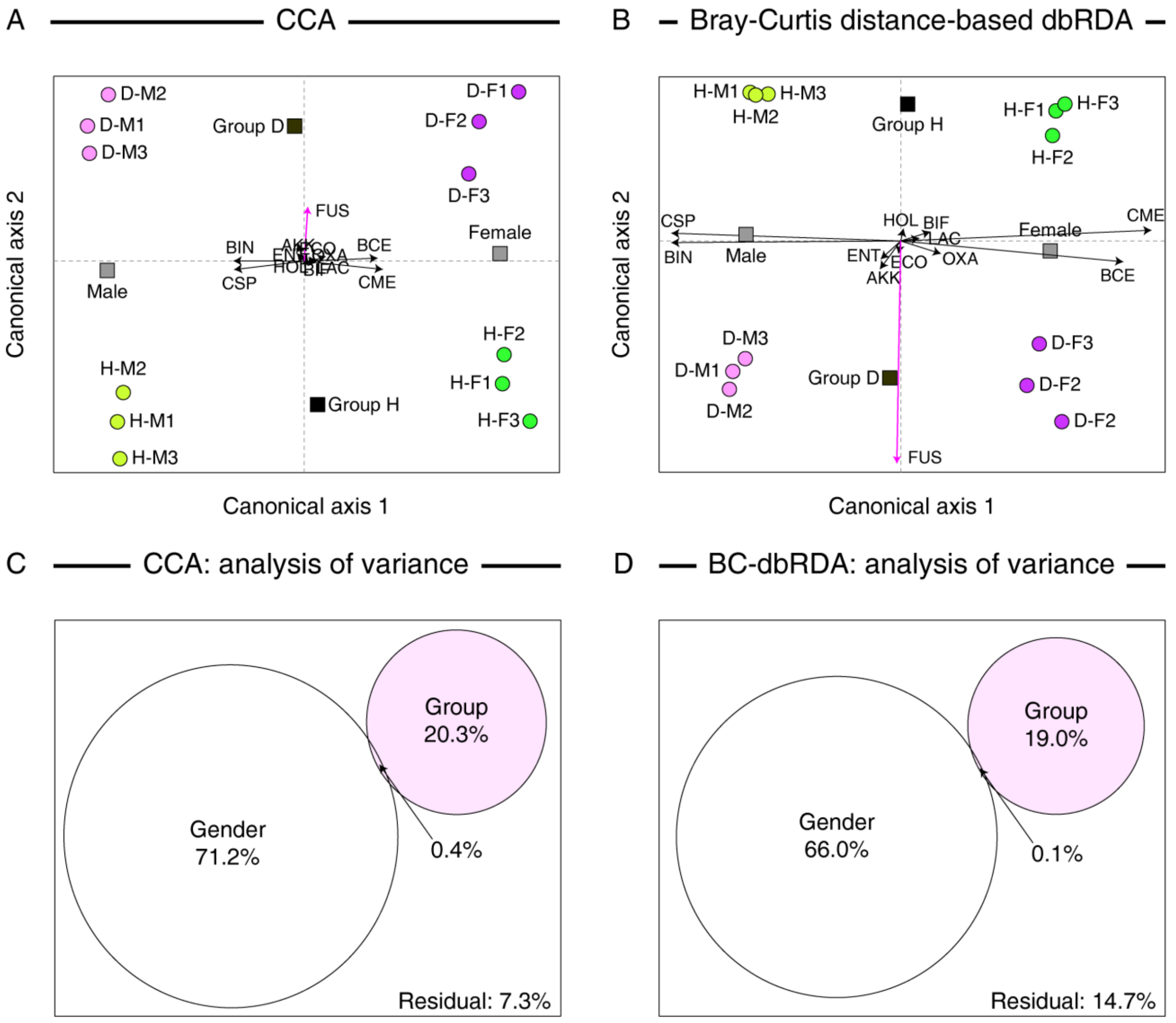

Supplementary Figure 2: Bray-Curtis distance based Principal response curves analysis performed on data from Supplementary File 2.

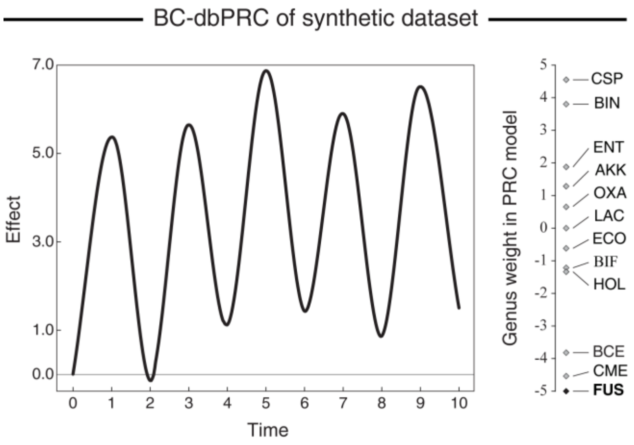

Supplementary File 1. Full numerical dataset and weighted UniFrac distance matrix used to run RDA and UF-dbRDA ordination analyses.

RDA input sample ID	Group	Gender	BIN	BCE	CSP	CME	AKK	ECO	OXA	BIF	LAC	ENT	HOL	FUS
H-M1	1	1	37	22	41	18	40	38	36	43	38	42	36	17
H-F1	1	2	19	36	23	44	36	43	43	43	44	39	37	21
H-M2	1	1	41	21	37	19	36	40	39	37	41	42	44	20
H-F2	1	2	21	41	20	39	36	42	36	40	38	39	44	22
H-M3	1	1	44	19	40	24	41	42	42	43	42	38	39	16
H-F3	1	2	21	41	19	43	41	38	43	38	40	40	41	17
D-M1	2	1	42	21	43	17	38	40	39	36	42	43	41	42
D-F1	2	2	20	42	17	41	44	36	43	43	39	43	39	44
D-M2	2	1	41	18	37	19	43	41	37	36	37	44	37	43
D-F2	2	2	23	42	22	40	38	43	40	41	42	41	36	42
D-M3	2	1	41	22	44	16	43	43	39	42	39	38	41	40
D-F3	2	2	22	40	22	39	39	44	44	43	44	40	44	39

dbRDA DM input

	D2gA1	D2gA2	D2gA3	D2gA4	D2gA5	D2gA6	D2gB1	D2gB2	D2gB3	D2gB4	D2gB5	D2gB6
D2gA1	0.000	0.080	0.061	0.063	0.041	0.045	0.112	0.121	0.124	0.109	0.107	0.107
D2gA2	0.080	0.000	0.077	0.074	0.065	0.071	0.127	0.130	0.141	0.118	0.128	0.104
D2gA3	0.061	0.077	0.000	0.037	0.062	0.053	0.102	0.124	0.126	0.101	0.107	0.092
D2gA4	0.063	0.074	0.037	0.000	0.063	0.053	0.101	0.118	0.125	0.091	0.101	0.084
D2gA5	0.041	0.065	0.062	0.063	0.000	0.036	0.126	0.132	0.140	0.119	0.119	0.109
D2gA6	0.045	0.071	0.053	0.053	0.036	0.000	0.118	0.126	0.135	0.112	0.116	0.103
D2gB1	0.112	0.127	0.102	0.101	0.126	0.118	0.000	0.060	0.050	0.039	0.044	0.054
D2gB2	0.121	0.130	0.124	0.118	0.132	0.126	0.060	0.000	0.041	0.059	0.050	0.067
D2gB3	0.124	0.141	0.126	0.125	0.140	0.135	0.050	0.041	0.000	0.065	0.051	0.078
D2gB4	0.109	0.118	0.101	0.091	0.119	0.112	0.039	0.059	0.065	0.000	0.043	0.039
D2gB5	0.107	0.128	0.107	0.101	0.119	0.116	0.044	0.050	0.051	0.043	0.000	0.052
D2gB6	0.107	0.104	0.092	0.084	0.109	0.103	0.054	0.067	0.078	0.039	0.052	0.000

Supplementary File 2. Full numerical dataset and weighted UniFrac distance matrix used to run PRC and UF-dbPRC analyses.

PRC input sample ID	Treatment	Time	BIN	BCE	CSP	CME	AKK	ECO	OXA	BIF	LAC	ENT	HOL	FUS
A-T0	0	0.1	22	38	21	39	37	41	38	43	40	36	42	41
A-T1	0	1	22	38	21	39	37	41	38	43	40	36	42	41
A-T2	0	2	22	38	21	39	37	41	38	43	40	36	42	41
A-T3	0	3	22	38	21	39	37	41	38	43	40	36	42	41
A-T4	0	4	22	38	21	39	37	41	38	43	40	36	42	41
A-T5	0	5	22	38	21	39	37	41	38	43	40	36	42	41
A-T6	0	6	22	38	21	39	37	41	38	43	40	36	42	41
A-T7	0	7	22	38	21	39	37	41	38	43	40	36	42	41
A-T8	0	8	22	38	21	39	37	41	38	43	40	36	42	41
A-T9	0	9	22	38	21	39	37	41	38	43	40	36	42	41
A-T10	0	10	22	38	21	39	37	41	38	43	40	36	42	41
B-T0	1	0.1	22	38	21	39	37	41	38	43	40	36	42	41
B-T1	1	1	41	19	41	19	44	37	40	40	40	37	39	33
B-T2	1	2	16	44	16	44	37	42	36	42	40	43	36	30
B-T3	1	3	36	24	41	19	37	44	38	44	38	44	36	20
B-T4	1	4	20	40	20	40	36	44	41	38	39	42	42	18
B-T5	1	5	39	21	44	16	42	43	41	39	43	43	36	21
B-T6	1	6	18	42	24	36	44	37	39	41	44	37	40	20
B-T7	1	7	39	21	41	19	42	41	40	43	38	40	43	18
B-T8	1	8	20	40	21	39	44	41	38	43	40	36	44	21
B-T9	1	9	43	17	41	19	36	36	41	37	40	38	44	19
B-T10	1	10	23	37	21	39	41	44	40	39	39	44	41	22

dbPRC DM input

	A-T0	A-T1	A-T2	A-T3	A-T4	A-T5	A-T6	A-T7	A-T8	A-T9	A-T10	B-T0	B-T1	B-T2	B-T3	B-T4	B-T5	B-T6	B-T7	B-T8	B-T9	B-T10
A-T0	0.000	0.000	0.000	0.000	0.000	0.000	0.000	0.000	0.000	0.000	0.000	0.000	0.131	0.125	0.207	0.207	0.232	0.197	0.207	0.170	0.221	0.189
A-T1	0.000	0.000	0.000	0.000	0.000	0.000	0.000	0.000	0.000	0.000	0.000	0.000	0.131	0.125	0.207	0.207	0.232	0.197	0.207	0.170	0.221	0.189
A-T2	0.000	0.000	0.000	0.000	0.000	0.000	0.000	0.000	0.000	0.000	0.000	0.000	0.131	0.125	0.207	0.207	0.232	0.197	0.207	0.170	0.221	0.189
A-T3	0.000	0.000	0.000	0.000	0.000	0.000	0.000	0.000	0.000	0.000	0.000	0.000	0.131	0.125	0.207	0.207	0.232	0.197	0.207	0.170	0.221	0.189
A-T4	0.000	0.000	0.000	0.000	0.000	0.000	0.000	0.000	0.000	0.000	0.000	0.000	0.131	0.125	0.207	0.207	0.232	0.197	0.207	0.170	0.221	0.189
A-T5	0.000	0.000	0.000	0.000	0.000	0.000	0.000	0.000	0.000	0.000	0.000	0.000	0.131	0.125	0.207	0.207	0.232	0.197	0.207	0.170	0.221	0.189
A-T6	0.000	0.000	0.000	0.000	0.000	0.000	0.000	0.000	0.000	0.000	0.000	0.000	0.131	0.125	0.207	0.207	0.232	0.197	0.207	0.170	0.221	0.189
A-T7	0.000	0.000	0.000	0.000	0.000	0.000	0.000	0.000	0.000	0.000	0.000	0.000	0.131	0.125	0.207	0.207	0.232	0.197	0.207	0.170	0.221	0.189
A-T8	0.000	0.000	0.000	0.000	0.000	0.000	0.000	0.000	0.000	0.000	0.000	0.000	0.131	0.125	0.207	0.207	0.232	0.197	0.207	0.170	0.221	0.189
A-T9	0.000	0.000	0.000	0.000	0.000	0.000	0.000	0.000	0.000	0.000	0.000	0.000	0.131	0.125	0.207	0.207	0.232	0.197	0.207	0.170	0.221	0.189
A-T10	0.000	0.000	0.000	0.000	0.000	0.000	0.000	0.000	0.000	0.000	0.000	0.000	0.131	0.125	0.207	0.207	0.232	0.197	0.207	0.170	0.221	0.189
B-T0	0.000	0.000	0.000	0.000	0.000	0.000	0.000	0.000	0.000	0.000	0.000	0.000	0.131	0.125	0.207	0.207	0.232	0.197	0.207	0.170	0.221	0.189
B-T1	0.131	0.131	0.131	0.131	0.131	0.131	0.131	0.131	0.131	0.131	0.131	0.131	0.000	0.059	0.104	0.109	0.115	0.082	0.096	0.066	0.112	0.086
B-T2	0.125	0.125	0.125	0.125	0.125	0.125	0.125	0.125	0.125	0.125	0.125	0.125	0.059	0.000	0.093	0.099	0.118	0.095	0.101	0.073	0.117	0.079
B-T3	0.207	0.207	0.207	0.207	0.207	0.207	0.207	0.207	0.207	0.207	0.207	0.207	0.104	0.093	0.000	0.041	0.045	0.060	0.044	0.069	0.058	0.047
B-T4	0.207	0.207	0.207	0.207	0.207	0.207	0.207	0.207	0.207	0.207	0.207	0.207	0.109	0.099	0.041	0.000	0.053	0.062	0.050	0.072	0.046	0.044
B-T5	0.232	0.232	0.232	0.232	0.232	0.232	0.232	0.232	0.232	0.232	0.232	0.232	0.115	0.118	0.045	0.053	0.000	0.059	0.048	0.081	0.054	0.056
B-T6	0.197	0.197	0.197	0.197	0.197	0.197	0.197	0.197	0.197	0.197	0.197	0.197	0.082	0.095	0.060	0.062	0.059	0.000	0.041	0.041	0.059	0.052
B-T7	0.207	0.207	0.207	0.207	0.207	0.207	0.207	0.207	0.207	0.207	0.207	0.207	0.096	0.101	0.044	0.050	0.048	0.041	0.000	0.048	0.054	0.045
B-T8	0.170	0.170	0.170	0.170	0.170	0.170	0.170	0.170	0.170	0.170	0.170	0.170	0.066	0.073	0.069	0.072	0.081	0.041	0.048	0.000	0.081	0.049
B-T9	0.221	0.221	0.221	0.221	0.221	0.221	0.221	0.221	0.221	0.221	0.221	0.221	0.112	0.117	0.058	0.046	0.054	0.059	0.054	0.081	0.000	0.069
B-T10	0.189	0.189	0.189	0.189	0.189	0.189	0.189	0.189	0.189	0.189	0.189	0.189	0.086	0.079	0.047	0.044	0.056	0.052	0.045	0.049	0.069	0.000

Supplementary File 3. R code and the instructions to run dbRDA and dbPRC analyses.

R script dependencies:

In order to run the provided scripts, the following R packages and input files are needed:

R packages:

1) vegan
2) phyloseq
3) ape

Input files:

1.) Matrix of response and explanatory variables (samples in rows, variables in columns, in .CSV format).
2.) Taxonomic map for the variables (from "Domain" to "OTU" level taxonomies, provided as .CSV).
3.) (Rooted) phylogenetic tree in Newick format (.TXT file).

```
###########################################################################

#Code for RDA and dbRDA
#Required packages
#library(phyloseq)
#library(ape)
#library(vegan)
rm(list = ls())

#Calculation of Weighted UniFrac distance matrix
#Read in the OTU and taxonomy input
otu.table<-read.csv("RDAinput.csv", sep = ",", header = TRUE, row.names = 1)
otu.table<-otu.table[,3:length(otu.table)]
tax.map<-read.csv("Taxmap.csv", sep = ",", header = TRUE)

#Create a phyloseq compatible OTU table
otu.table<-t(otu.table)
otu.table<-data.matrix(otu.table, rownames.force = TRUE)
tax.map<-as.matrix(tax.map)
phyloseq.otu.table<-otu_table(otu.table, taxa_are_rows = TRUE)
class(phyloseq.otu.table) #should return "phyloseq"
class(tax.map) #should return "matrix"

#Read in the phylogenetic tree matrix
phy.tree<-read_tree("Phylotree.txt")
phy.tree<-root(phy.tree, outgroup = "AKK", resolve.root = TRUE) #Specific to test dataset
```

```
# plot(phy.tree)

#Merge OTU table, taxonomy map, and phylogenetic tree for UniFrac calculation
phylo.set<-merge_phyloseq(phy.tree, phyloseq.otu.table, tax.map)

#Calculate Weighted UniFrac distance matrix
wUF.dm<-UniFrac(phylo.set, weighted = TRUE, fast = TRUE)
wUF.table<-as.matrix(dist(wUF.dm))

#Save distance matrix to file
write.table(wUF.table, file = "wUFdm.csv", sep = ",")

#Calculate Principal Coordinates and plot
wUF.pcoa<-pcoa(wUF.dm)
wUF.pcoa.plot<-biplot(wUF.pcoa)

#Calculation of UniFrac distance and non-distance based Redundancy Analysis
#Read in RDA input
parent<-read.csv("RDAinput.csv", sep = ",", header = TRUE, row.names = 1)

#Set objects
response<-parent[,3:length(parent)]
group<-as.factor(parent[,1])
gender<-as.factor(parent[,2])

#Calculate non-distance based RDA
RDA<-rda(response ~ group+gender)
plot(RDA, main = "RDA")
RDA
anova.cca(RDA, by="terms")

#Calculate distance based RDA using Weighted UniFrac distance matrix
dbRDA<-capscale(wUF.dm ~ group+gender, env = parent[,1:2], comm = response)
plot(dbRDA, main = "dbRDA")
dbRDA
anova.cca(dbRDA, by="terms")

#Any output can be written to comma-separated file using the below command.
#write.table(object to be written to file, file = "filename", sep = ",")

##########################################################################

#Code for PRC and dbPRC
#Required packages
#library(vegan)
#library(phyloseq)
```

```
#library(ape)
rm(list = ls())

#Read in dataset
parent<-read.csv("PRCinput.csv", sep = ",", header = TRUE, row.names = 1)
response<-parent[,3:length(parent)]
time<-as.factor(parent[,2])
treatment<-as.factor(parent[,1])

#Set up objects and matrices
y <- deparse(substitute(response))
x <- deparse(substitute(treatment))
z <- deparse(substitute(time))

#Create formulas and design matrices
fla <- as.formula(paste("~", x, "+", z))
mf <- model.frame(fla, response, na.action = na.pass)
fla.zx <- as.formula(paste("~", z, ":", x))
fla.z <- as.formula(paste("~", z))
X = model.matrix(fla.zx, mf)[, -c(seq_len(nlevels(time) + 1))]
Z = model.matrix(fla.z, mf)[, -1]

#Calculation of non-distance based PRC
#Non-distance based RDA
RDA <- rda(response ~ X + Condition(Z))

#Conversion of RDA to PRC compatible format
RDA$terminfo$xlev = list(levels(time), levels(treatment))
names(RDA$terminfo$xlev) = c(paste(z), paste(x))
RDA$call <- match.call()
class(RDA) <- c("prc", class(RDA))
plot(RDA, main = "PRC")

#Store species weights
RDA.sp<-linestack(scores(RDA, ch = 1, display = "species", scaling = 3))
RDA.sp<-as.matrix(RDA.sp)
row.names(RDA.sp) = rownames(scores(RDA, display = "species"))
colnames(RDA.sp) = "Weight"

#Calculation of Weighted UniFrac
#Read in the OTU community set
otu.table<-read.csv("PRCinput.csv", sep = ",", header = TRUE, row.names = 1)
otu.table<-otu.table[,3:length(otu.table)]

#Create phyloseq compatible OTU and taxonomy tables
otu.table<-t(otu.table)
```

```
tax.map<-read.csv("Taxmap.csv", sep = ",", header = TRUE)
otu.table<-data.matrix(otu.table, rownames.force = TRUE)
tax.map<-as.matrix(tax.map)
phyloseq.otu.table<-otu_table(otu.table, taxa_are_rows = TRUE)
class(phyloseq.otu.table)
class(tax.map)

#Read in the phylogenetic tree matrix
phy.tree<-read_tree("Phylotree.txt")
phy.tree<-root(phy.tree, outgroup = "AKK", resolve.root = TRUE) #Specific to test dataset
plot(phy.tree)

#Merge OTU table, taxonomy map, and phylogenetic tree into phyloseq object
phylo.set<-merge_phyloseq(phy.tree, phyloseq.otu.table, tax.map)

#Calculate Weighted UniFrac distance matrix
wUF.dm<-UniFrac(phylo.set, weighted = TRUE, fast = TRUE)
wUF.table<-as.matrix(dist(wUF.dm))

#Save distance matrix to file
write.table(wUF.table, file = "wUFdm.csv", sep = ",")

#Calculation of UniFrac distance based PRC
dbRDA <- capscale(wUF.dm ~ X + Condition(Z), data = parent[,1:2], comm = response)

#Conversion of dbRDA to PRC compatible format
dbRDA$terminfo$xlev = list(levels(time), levels(treatment))
names(dbRDA$terminfo$xlev) = c(paste(z), paste(x))
dbRDA$call <- match.call()
class(dbRDA) <- c("prc", class(dbRDA))
plot(dbRDA, main = "dbPRC")

#Store species weights
dbRDA.sp<-linestack(scores(dbRDA, ch = 1, display = "species", scaling = 3))
dbRDA.sp<-as.matrix(dbRDA.sp)
row.names(dbRDA.sp) = rownames(scores(dbRDA, display = "species"))
colnames(dbRDA.sp) = "Weight"
```
